# Through Their Lens: Using Visual Methods to Illuminate the Lived Experiences of Diverse Older Adults

**DOI:** 10.1093/geroni/igaa057.2580

**Published:** 2020-12-16

**Authors:** Jarmin Yeh, Tam Perry

## Abstract

Visual methods, like photovoice and photo-elicitation, have attracted modest attention in gerontological inquiry with diverse and vulnerable community-dwelling older adults. Visual methods are based on the idea of inserting images, produced by informants or not, into research interviews, allowing informants to be the experts of knowledge and meaning-making while the researcher becomes the student. The empowerment of informants as subject-collaborators in the research process is a distinctive feature of visual methods. Benefits include revealing unique insights into diverse phenomena by evoking elements of human consciousness, feelings, and memories that words may not easily express and surveys may not easily capture. This symposium presents qualitative research using visual methods to illuminate the lived experiences, voices, and perspectives of diverse and vulnerable older adults living in New Jersey, Connecticut, and California. Reyes’ research critiques how the operationalization of mainstream notions of civic participation becomes exclusionary and provides a more inclusive understanding of how civic participation is enacted and performed through the practices of Latinx and African American older adults living in New Jersey. Versey’s research with homeless older adults subverts the attention often focused within cities by interrogating the meaning of place with informants whose needs and desires are often overlooked or obscured by residing in a small, rural town in central Connecticut. Yeh’s research on aging in place inequalities chronicles the everyday lives of housed and unhoused older San Franciscans to reveal their tactics for negotiating a moving tension between the daily interiority of identity and contingencies of a changing environment. Qualitative Research Interest Group Sponsored Symposium.

